# Dr. Beddoes's Plan for Trying Acids, &c

**Published:** 1801-08

**Authors:** T. Beddoes


### *To the Editors of the Medical and Physical Journal.*

GENTLEMEN,

I Have been silently preparing to bring the too long depending question concerning acids in Syphilis to a decision, according to the terms of my challenge to Mr. Pearson. I am impatient that it should be known to the whole public, whether careless management, with foul play and an insufficient range of of powers, occafioned the lofs of many lives, and the deftruction of many conftitutions, which acids would have faved.

I requeft you to infert the enclofed paper, or a fufficient abftract of it, in your valuable Journal. I have only to add, that I am ready to communicate with any members of the medical profeffion, on the regulations to be adopted with regard to patients, and their reception at the Pneumatic Inftitution.

I am, &c.

*July* 18, 1801. T. BEDDOES.

---

*Plan of a Public Scrutiny of certain Medicines, lately proposed as anti-venereal, at the Pneumatic Institution near Bristol.*

THE idea of a public trial of acids in the venereal difeafe feems to have been generally approved; and I have now the fatisfaction of making it known, that the means of fetting the fcheme on foot are in my poffeffion.

In favour of the meafure itfelf many reafons might be adduced. I fhall truft to the three following as decifive;—*the desirableness of a new anti-venereal remedy*; *the difficulty of satisfying the public mind by individual teftimony*; *and the little disposition manifested by the profession at large, to take the trouble necessary to decide the question.*

I. A greater benefit could fcarcely be conferred on human fociety than the difcovery of a new remedy for the venereal difeafe. This is acknowledged by all parties. But however ftrongly men, experienced in medicine, may feel the urgency of the exifting neceffity, no general terms can convey an idea of it to others. The relation of particular facts would far exceed the bounds of a profpectus, or do the fubject the greateft injuftice. Where the conftitution, from early mifmanagement, becomes thoroughly impregnated with the venereal poifon, mercury not unfrequently fails, or, by the ufe of this fevere medicine, the patient dearly earns a fhort refpite from his daily and nightly tortures. This is more commonly the lot of the ignorant and the poor, of whom, after they have in vain tried hofpital practice and advertifed medicines, it remains untold in what corner they hide themfelves to be preyed upon by their unrelenting diforder. But mercury (which muft ftill be allowed a ftation in the foremoft rank among the articles of the *Materia Medica*) proves alfo the occafional fcourge of the wealthy and the knowing. In particular inftances, however fkilfully managed, it is ineffectual, or it aggravates the complaint. When it anfwers the immediate end, it gives rife to diforders that either foon arrive at a fatal termination, or blaft the the ſpring of life, induce premature decay, and afflict advancing years with evils not their own. The tranſmiſſion of the proper venereal diſeaſe is diſputed. But what concerns ſociety full as much is indiſputable; namely, the tranſmiſſion of morbid tendencies, engendered by its reputed *sole specific*. Hence the ſpectacle of parents, condemned to witneſs their youthful imprudencies, viſited upon their children, too often occurs to thoſe to whom the interior condition of human beings is laid open without diſguiſe.

In corroboration of this ſtatement I may appeal to our cities, our univerſities, the army and the navy. Wherever youth feels and indulges its ordinary propenſities, there mercury lays the foundation of evils, the contemplation of which ought ſurely to produce ſomething beyond barren horror or commiſeration.

II. Could the maſs of teſtimony either for or againſt the acids be annihilated, that on the other ſide muſt, from its multiplicity and ſtrength, decide the public opinion. What therefore can be expected from freſh acceſſions of private teſtimony? If *success* is announced, how eaſy to ſuppoſe a miſtake in regard to the diſeaſe, or to find ſome other method of explaining away the fact! *Failure* may be equally imputed to miſmanagement. For nothing is ſo eaſy as to adminiſter acids with ſafety to the patient, but without effect on the diſeaſe. The diſpute therefore will be left as it ſtands. Nor will the negligent, the luke-warm, the biaſſed, and the envious be at a loſs for unſucceſsful reports, till they ſhall feel the controul of general opinion, eſtabliſhed upon experiments beyond the reach of cavil.

III. It has been ſurmiſed that the maſs of atteſtations, lately collected, and the accumulated proofs of a proper ſalivating power in acids, would ſet innumerable enquirers at work. A conſiderable time has however elapſed, and there are tokens enough of a communicative diſpoſition among the Faculty; yet no ſingle ſymptom of the predicted ardour of inveſtigation has tranſpired.

Further reaſons will hardly be required. Otherwiſe I might go on to explain what a blow empirical impoſture would receive from the aſcertainment of antivenereal power in new ſubſtances; and how much our medical logic would be improved. For it is hardly conceivable to what a degree the excluſive opinion, commonly entertained, narrows our views of organic ſuſceptibility. As if it were poſſible that any conſiderable proportion of the medicinal treaſures of Nature could be known, when, in the courſe of ages, no enterpriſe of diſcovery worth mentioning has been undertaken!

It

It remains that the Inquiry be unexceptionably conducted; fo that Europe may be no longer defrauded of that benefit from acids which India is unanimoufly attefted to enjoy. All doubt as to the nature of the cafes muft be obviated. Hence it fhould be a rule, to receive no patient without a ftatement figned by at leaft three phyficians or furgeons. And every patient muft be infpected, on his reception and during the treatment, by practitioners on the fpot.

But the public muft be fatisfied that the medicines alleged are adminiftered, and no others. A perfon, worthy of confidence, muft therefore be engaged to fuperintend the treatment. And although this perfon, in common with others, may exercife his judgment upon the cafes, nothing will reft on his fingle opinion.

The author of this paper having actually tried many varieties of acid medicines, and formed other combinations in his mind, propofes to *direct* the treatment. But he will hold no communication with any patient but in the prefence of the fuperintendant.—To him, in his fituation, celebrity in any other common diforder would be far more lucrative. But as the confignment to his care of the papers, relative to a practice that was originally promoted by his crude fpeculations, firft engaged him in the queftion, fo a fenfe of its importance now induces him to fubmit to exertions in bringing it to an iffue. He is convinced that many more difficult inveftigations may be propofed in medicine, but not one more immediately ufeful.

He ftill abides by the opinion expreffed in his letter to Mr. Pearfon, "*that the patients must be persons of regular conduct, and concerning whom we may be able to procure information at a considerable distance of time.*" (Communications, p. lxi.)

It will be neceffary perhaps to take fome pledge for the perfeverance of the patient, to whom it fhould be previoufly explained that he is to be treated on a plan fuppofed on fuch and fuch grounds efficacious, and lefs trying to the conftitution than the mercurial. As foon as it fhould be deemed improper to continue the new plan, the old one will be adopted.

Patients with the proper certificate would be admitted to the number of twelve; and though little ftrefs would be laid on out-patients, yet any number of thefe would be treated.

One day in the week, at certain hours, the houfe would be open to all vifitors whatfoever. The practitioners who choofe to obferve and atteft thefe experiments muft have the neceffary accefs to the patients.

The firft object in view is to fatisfy the public if certain fubftances not mercurial, are or are not remedies for the confirmed firmed venereal diſeaſe. But I could alſo wiſh to continue the trials till ſome judgment can be formed whether, if they be antivenereals, they equal mercurial preparations in virtue.

With the neceſſary co-operation on the part of the profeſſion to ſupply patients and obſerve the treatment, I truſt that with what I may afford from my own purſe, added to the remainder of the ſubſcriptions to the Pneumatic Inſtitution (which the committee is willing to have expended in this manner) and to ſome contributions for this expreſs purpoſe, I ſhall be able to ſolve the firſt queſtion.

Whether I ſhall have ability or funds to ſolve the ſecond, will depend upon the opulent part of the public. That they will feel how deeply they are intereſted in the ſubject, I cannot foreſee. But, for helping to render this enquiry more full and ſatisfactory, thoſe among their ſons, who are liable to have their conſtitutions impaired or deſtroyed by the operation of mercury, would owe them greater obligations than for a large patrimony. And (if on ſuch a ſubject one may ſpeak the truth in defiance of falſe delicacy) they will not leſs eſſentially ſerve their daughters by ſaving their future huſbands from the ravages of the ſame remedy.

*Those who may incline to support this scheme, are desired to forward their subscriptions to Messrs. Couts and Co. Bankers, Strand, London; or to Messrs. Savery and Pugh, Bankers, Bristol.*

