# Erratum

**Published:** 1801-09

**Authors:** 


ERRATUM.

*In Dr. Beddoes's Letter, in our last Number, p.* 165, *before* occasioned, *insert* has not.

